# Logical Observation Identifiers Names and Codes (LOINC^®^) Applied to Microbiology: A National Laboratory Mapping Experience in Taiwan

**DOI:** 10.3390/diagnostics11091564

**Published:** 2021-08-28

**Authors:** Chih-Yang Yeh, Syu-Jyun Peng, Hsuan Chia Yang, Mohaimenul Islam, Tahmina Nasrin Poly, Chien-Yeh Hsu, Stanley M. Huff, Huan-Chieh Chen, Ming-Chin Lin

**Affiliations:** 1 Graduate Institute of Biomedical Informatics, College of Medicine Science and Technology, Taipei Medical University, Taipei 11031, Taiwan; d610104001@tmu.edu.tw (C.-Y.Y.); lovejog@tmu.edu.tw (H.C.Y.); d610106004@tmu.edu.tw (M.I.); d610108004@tmu.edu.tw (T.N.P.); 2 Professional Master Program in Artificial Intelligence in Medicine, College of Medicine, Taipei Medical University, Taipei 11031, Taiwan; sjpeng2019@tmu.edu.tw; 3 International Center for Health Information Technology (ICHIT), Taipei Medical University, Taipei 11031, Taiwan; 4 Research Center of Big Data and Meta-Analysis, Wan Fang Hospital, Taipei Medical University, Taipei 116, Taiwan; 5 Department of Information Management, National Taipei University of Nursing and Health Science, Taipei 11219, Taiwan; cyhsu@ntunhs.edu.tw; 6 Master Program in Global Health and Development, Taipei Medical University, Taipei 11031, Taiwan; 7 Department of Biomedical Informatics, School of Medicine, University of Utah, Salt Lake City, UT 84132, USA; stan.huff@imail.org; 8 Department of Biomedical Informatics, Intermountain Healthcare, Murray, UT 84107, USA; 9 Department of Neurosurgery, Taipei Medical University-Wan Fang Hospital, Taipei 116, Taiwan; dissector@gmail.com; 10 Taipei Neuroscience Institute, Taipei Medical University, Taipei 11031, Taiwan; 11 Department of Neurosurgery, Shuang Ho Hospital, Taipei Medical University, New Taipei City 23561, Taiwan

**Keywords:** LOINC laboratory test, RELMA, automated mapping, electronic health record

## Abstract

Background and Objective: Logical Observation Identifiers Names and Codes (LOINC) is a universal standard for identifying laboratory tests and clinical observations. It facilitates a smooth information exchange between hospitals, locally and internationally. Although it offers immense benefits for patient care, LOINC coding is complex, resource-intensive, and requires substantial domain expertise. Our objective was to provide training and evaluate the performance of LOINC mapping of 20 pathogens from 53 hospitals participating in the National Notifiable Disease Surveillance System (NNDSS). Methods: Complete mapping codes for 20 pathogens (nine bacteria and 11 viruses) were requested from all participating hospitals to review between January 2014 and December 2016. Participating hospitals mapped those pathogens to LOINC terminology, utilizing the Regenstrief LOINC mapping assistant (RELMA) and reported to the NNDSS, beginning in January 2014. The mapping problems were identified by expert panels that classified frequently asked questionnaires (FAQs) into seven LOINC categories. Finally, proper and meaningful suggestions were provided based on the error pattern in the FAQs. A general meeting was organized if the error pattern proved to be difficult to resolve. If the experts did not conclude the local issue’s error pattern, a request was sent to the LOINC committee for resolution. Results: A total of 53 hospitals participated in our study. Of these, 26 (49.05%) used homegrown and 27 (50.95%) used outsourced LOINC mapping. Hospitals who participated in 2015 had a greater improvement in LOINC mapping than those of 2016 (26.5% vs. 3.9%). Most FAQs were related to notification principles (47%), LOINC system (42%), and LOINC property (26%) in 2014, 2015, and 2016, respectively. Conclusions: The findings of our study show that multiple stage approaches improved LOINC mapping by up to 26.5%.

## 1. Introduction

Logical Observation Identifiers Names and Codes (LOINC^®^) is the global standard terminology for identifying and describing laboratory examinations [1,2]. The Regenstrief Institute, a US non-profit organization, developed and has maintained LOINC since 1994, and it is currently adopted in more than 165 countries worldwide [3,4]. The adoption of LOINC in hospitals expedites the exchange of laboratory and clinical data among various information systems, providers, and individuals. The logic behind developing the LOINC code was to facilitate interoperability between systems (i.e., electronic health records (EHRs) and laboratory information systems (LISs)) for sharing laboratory results by reducing complex mapping [4,5,6]. Hospitals were previously facing immense problems sharing data locally and globally due to universally recognized and specialized terminologies before the initiation of LOINC [7,8]. Each country and hospital developed its own local codes to record laboratory test findings, which created potential challenges for laboratory test ordering as well as reporting, interpreting, comparing, and sharing [9,10,11].

**Figure 1:**
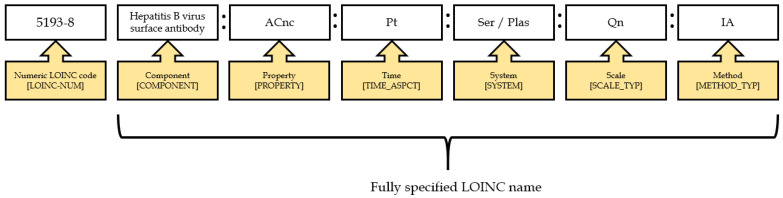
An example of a LOINC name and code.

In recent decades, the scope of LOINC content has significantly increased, extending to clinical and non-clinical observations, such as vital signs, echocardiography, obstetric ultrasound, pulmonary ventilator management, Glasgow coma score, gastro-endoscopic procedures, and billing [12,13,14,15]. Taiwan has developed an electronic laboratory reporting (ELR) system and adopted and used standardized terminologies since 2006. Liu et al. [16] reported that the mapping ratio of the national health insurance (NHI) codes to LOINC codes was low (17%) and that the NHI codes were too generic (imprecise) to map exactly to the LOINC codes. Appropriate coding from NHI codes to LOINC codes requires support from both physicians and laboratory technologists, which is labor intensive and could hamper the performance of current operating procedures. However, Lee et al. [17] developed a multi-part matching strategy algorithm to improve mapping quality and reduce manual efforts. The performance of the automated model was better than the Regenstrief LOINC Mapping Assistant (RELMA) and Lab Auto Mapper (LAM) in terms of recall (86% vs. 78%) and precision (75% vs. 69%). There are several challenges when mapping Taiwanese local codes to LOINC codes, including completeness (insufficient information), coding variance (use of different LOINC codes for the same test), correctness (lack of LOINC terminology knowledge), local issues (too many codes for specimens and methodology), and literacy of standardized terminology (lack of LOINC expertise). According to the LOINC naming convention, every laboratory test name is composed of six parts: component, property, timing, system, scale, and method (Figure 1) [18].

Several studies have already suggested possible ways to overcome LOINC mapping. Lin et al. [19] suggested using more specific naming conventions for local descriptions, providing better training, and developing automated mapping tools. Dixon et al. [20] highlighted the importance of developing enhanced RELMA functions, mapping local terms to LOINC codes effectively. There are few previous studies that describe the process of mapping local test names to LOINC in detail. The objective of our study was to improve LOINC mapping quality, which is separated into three specific aims: (1) to improve LOINC mapping performance in the ELR system through training; (2) to analyze the local codes and standardize the mapping patterns or characteristics for 20 pathogens; and (3) to describe the local issues for requesting new LOINC codes for coverage of pathogen terms.

## 2. Materials and Methods

### 2.1. Study Background

**Figure 2:**
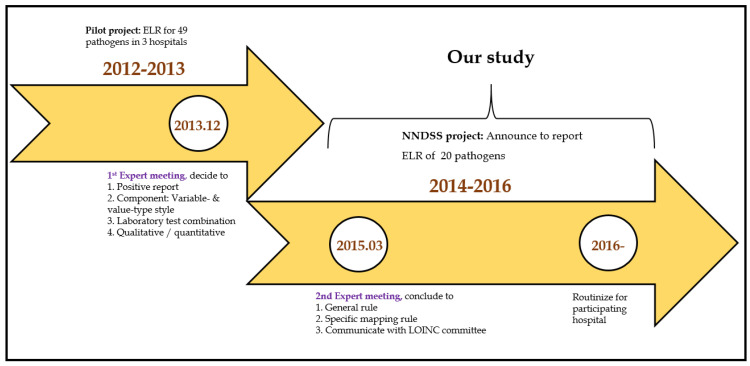
Timeline for NNDSS.

In 2012, the Taiwan Center for Disease Control (TCDC) conducted a pilot study to investigate how to implement ELR in Taiwan. A total of 49 pathogens’ data were collected from three hospitals and utilized RELMA for LOINC mapping. The primary objectives were: (1) to develop a LOINC mapping tool for 49 pathogens, which could reduce the burden of the LOINC mapping process; (2) to hold a training course to improve the mapping consistency; (3) to standardize the laboratory tests results using the Systematized Nomenclature of Medicine—Clinical Terms (SNOMED CT) for analysis; (4) to manage the mapping table of local codes to LOINC codes for version updates (Figure 2).

In 2013, the TCDC organized a meeting of experts to decide how to map local laboratory test codes to the LOINC terminology. All experts provided suggestions to: (a) establish a validation cycle for LOINC mapping; (b) collect positive laboratory results for reportable diseases as specified by the US Centers for Disease Control and Prevention (CDC); (c) take note of the difference of variable- and value-type styles; and (d) include all laboratory tests combinations for a specific condition (notifiable disease) and all the laboratory tests results as narrative or numerical data.

### 2.2. Development of the NNDSS Study Plan

**Figure 3:**
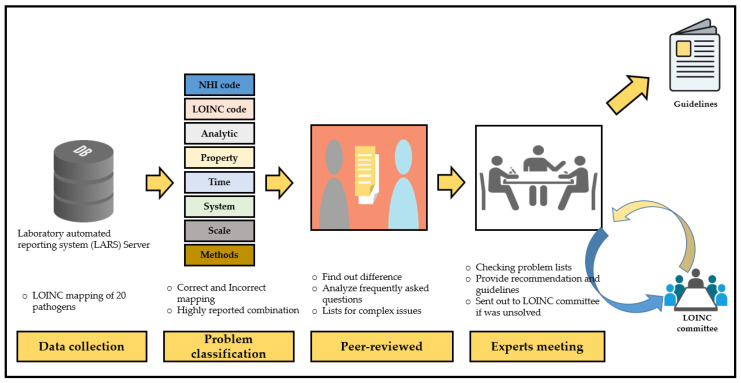
The entire process of the NNDSS project.

The TCDC again organized a meeting of experts, consisting of medical informaticians, laboratory experts, and CDC specialists to set general goals for encouraging and improving LOINC mapping (Figure 3).

In the strategy meeting, we decided on a multi-stage approach to implement NNDSS. Using this approach, we can observe or estimate the general condition in different types of hospitals, such as public, private, centers, regional, district, and affiliated hospitals. We gradually invited the nationwide representative hospitals first, and the regional hospital were in the second choice. Afterward, we invited the branches of hospitals, which are part of a multi-hospital system.

**Figure 4:**
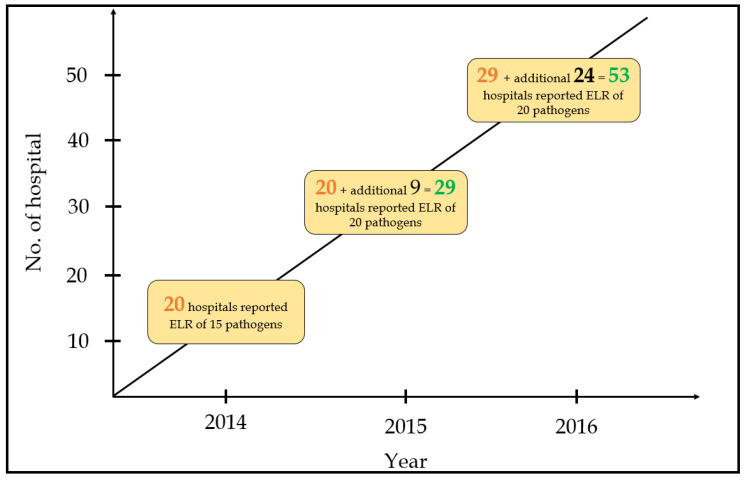
Number of hospitals and reported pathogens during the three-year study period.

The NNDSS project started in 2014 with 20 hospitals. Financial support was proposed to all participating hospitals (Supplementary Table S1), who were requested to use LOINC codes for notification of 20 pathogens, including nine bacteria and 11 viruses. Regarding bacteria, the TCDC mainly focused on tuberculosis, childhood diseases, and zoonotic foodborne diseases. Concerning viruses, the TCDC focused on influenza, chronic hepatitis, and childhood illnesses. In 2015, another nine hospitals participated in the NNDSS and reported LOINC codes for 20 pathogens. Ultimately, an additional 22 hospitals joined the NNDSS and reported LOINC codes for 20 pathogens (Figure 4).

#### 2.2.1. Data Collection and Problems Classification

Data were collected from all participating hospitals, including data reported concerning viral hepatitis, mycobacterium, antigen or antibody tests for non-mycobacterium, antigen or antibody tests for non-viral hepatitis, and viral/bacterial cultures. The mapping problems were identified by reviewers and classified as correct and incorrect mapping, similar to the LOINC System in incorrect terms. The reviewers also took the necessary steps to correct problem lists. They evaluated reported data based on the following criteria:

(a) Correctness—Each part matched to the original meaning of the test based on LOINC’s six parts;
(b) Usefulness—Mapped with suitable LOINC codes for those tests;
(c) Completeness—All required information was accurately represented in the mapping of the tests;
(d) Consistency—Ensured all of the laboratory test combinations had their unique meaning.

#### 2.2.2. Peer Review

Experts reviewed the mapping differences of those cases and suggested how to correct them. The expert panel consisted of LOINC specialists:

(a) Medical informaticians: LOINC experts who were familiar with the six axes (component, property, time, system, scale, and method) of the LOINC model and suggested the LOINC mapping rule, e.g., LOINC for a manual count of white blood cells in cerebral spinal fluid specimen is presented by LOINC code 806-0 (Leukocytes: NCnc: Pt: CSF: Qn: Manual count). Here is the breakdown of the fully specified name for LOINC code 806-0—component: Leukocytes, property: NCnc (number concentration), time: Pt (point in time), system (specimen): CSF (cerebral spinal fluid), scale: Qn (quantitative), method: manual count (Supplementary Table S1).
(b) Laboratory experts: Medical technologists who understand the laboratory tests meaning in the clinical laboratory and clarify the test terms in the laboratory information system, e.g., in HBsAg, they use the serum to analyze, but they use “Blood” in the LIS.
(c) Officials from the TCDC: TCDC specialists who established the NNDSS reporting rules and collected the laboratory test data of disease notifications from all large hospitals in Taiwan, e.g., in *Mycobacterium tuberculosis* complex, they collected the positive reports of the acid-fast stains, which are used as the preliminary mycobacterium culture for the purpose of prevention.

#### 2.2.3. Consensus Formation

Recommendations were provided based on the summary of errors report (Table 1). First, proper and meaningful suggestions were given for the error pattern in the frequently asked questions (FAQs). Second, a meeting was organized if the error pattern seemed to be more complicated to resolve. Third, if experts were unable to determine the local issue error pattern, we requested the LOINC committee to examine the problems (Figure 2).

## 3. Results

### 3.1. Homegrown vs. Outsourcing

There were 53 hospitals included in our study. Of these 53 hospitals, 26 (49%) used homegrown (local laboratory people did the mapping) LOINC mapping and 27 (51%) used outsourced LOINC mapping. There were no statistically significant differences in LOINC mapping between homegrown and outsourced (82.6% vs. 82.0%) (Table 2). In the homegrown system, the participants who joined the project in 2016 (early participants), mapped LOINC codes more than the participants who joined the project between 2014 and 2015 (late participants) (91.3% vs. 80.0%). Moreover, the performance of LOINC mapping in early participants was greater than the late participants while using outsourcing (84.9% vs. 76.3%). Three (A, B, C) out of six vendors provided most of the outsourcing (Supplementary Table S2). Table 2 shows the LOINC mapping completeness from two difference sources.

### 3.2. Performance Evaluation

**Figure 5:**
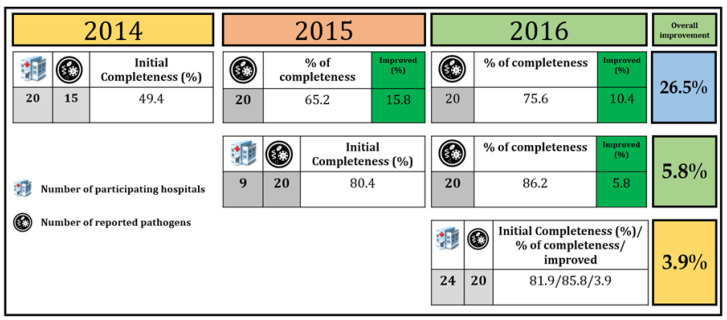
LOINC mapping performance comparison among 53 hospitals.

We evaluated the performance of LOINC mapping each year. Hospitals that joined the project in 2014 had improvements in LOINC mapping by 26.5% compared to hospitals that joined in 2015 and 2016 (5.8 and 3.9%, respectively) (Figure 5). Hospitals that joined earlier had more significant improvement than hospitals that joined later. This is because they received more suggestions and continuous feedback from experts to improve their LOINC coding.

### 3.3. Experience vs. Inexperience

Participants who had previous user experience in LOINC coding showed greater completeness than participants who had no experience in LOINC coding (Table 3). There was a statistically significant difference in LOINC mapping completeness between groups (Mann–Whitney *U* test: *p*-value = 0.037).

### 3.4. Analyses of Frequently Asked Questions (FAQs)

**Figure 6:**
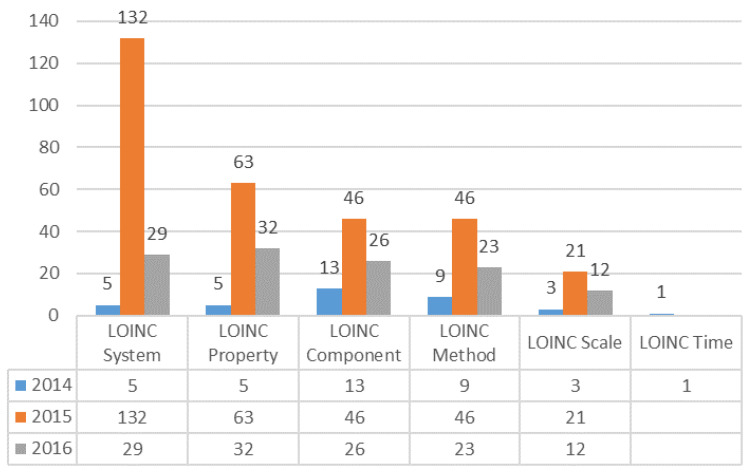

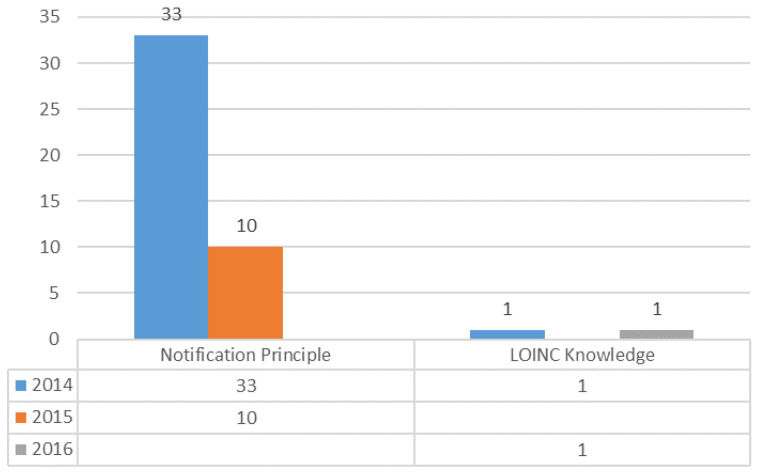
Analysis of frequently asked questions (FAQs) regarding LOINC mapping.

We collected the questions concerning LOINC coding difficulties during the three year study period and grouped them into three categories: notification principle, LOINC knowledge, and LOINC domain (component, property, time, system, scale, method). In 2014, there were 70 questions reported in the ELR. The main issues were about the notification principle (47%), which is related to reported format, laboratory result presentations, and ways to present the results. In 2015, the number of FAQs had increased to 318 and a higher number of questions were related to the LOINC System (42%). In 2016, 123 questions concerned LOINC mapping, and all questions were related to LOINC domains, such as LOINC property (26%), LOINC system (23%), LOINC component (21%), and LOINC method (19%) (Figure 6).

### 3.5. Evaluation and Suggestions for LOINC Mapping

The evaluation of LOINC mapping was classified into three steps: (a) implementation of ELR; (b) audit of LOINC mapping; and (c) evaluated reported data (Table 4). We categorized LOINC mapping error into different types of items. Finally, we suggested LOINC mapping guidelines based on those errors. For example, to participants who had general errors by design (variable-style names or value-style names) and specific topic issues for each disease, i.e., the rapid influenza immunoassay for the diagnosis of influenza virus A and B, we suggested to the mapping of LOINC 72367-6. For the test for group B streptococcus in women 35 to 37 weeks pregnant, we suggested users map LOINC 72607-5.

Finally, we checked the accuracy of mapping and compared previous mappings to new mappings (Table 5). We observed several codes affected when participants migrated LOINC Version 2.50 to 2.64. We noticed some axes were changed instead of creating a new LOINC code (old codes were deprecated). Some laboratory tests were mapped to LOINC code with no suitable axes. In order to retian consistency, our guidelines suggested some laboratory tests to map fixed axes with no LOINC code in temporary.

## 4. Discussion

In our study, we assessed the LOINC mapping performance for 20 pathogens in 53 hospitals in Taiwan and provided mapping suggestions for the error pattern in the FAQs. The findings of our study show that mapping performance increased by up to 26.5%; however, there were no significant differences in LOINC mapping between homegrown and outsourcing (82.6% vs. 82.0%). It was also notable that the mapping rate was higher among the hospitals with previous experience of LOINC coding than that of inexperienced hospitals. The most frequently asked question in 2014 was associated with notification principles; LOINC system and LOINC domain related questions were asked about more in 2015 and 2016, respectively (Table 6). Our study also shows that suggestions from experts and training courses lead to more consistent use of LOINC codes with less complexity and more accuracy.

Until now, most hospitals have used their own local and idiosyncratic codes to identify laboratory tests [21]. For example, one hospital would record and identify serum sodium with the code “C1231” and another hospital with the code “SNA” [9]. Each hospital has its own unique code for each laboratory test, which always makes it difficult to exchange information between hospitals. LOINC has provided a universal identifier for each test and has provided multiple opportunities to map laboratory tests [22]. LOINC was initially developed more than 20 years ago and approximately 36,000 registered users from more than 165 countries are using it, and many countries have officially adopted it as a national standard [23,24].

Despite the benefits of using LOINC codes for laboratory test records and exchange, mapping local terms to standard terminologies such as LOINC is complex and labor intensive [25,26,27,28]. Appropriate mapping with LOINC depends on domain knowledge and knowledge of the target vocabulary standard. Although laboratory technologists and physicians always have a good knowledge of laboratory tests, they may lack the knowledge of how to map local laboratory test codes to LOINC codes [4,29,30,31]. Moreover, local test coding has always lacked information to successfully map all laboratory tests to standard terminology [32]. Previous studies developed various algorithms to automatically map local codes to LOINC codes with great efficiency and minimum human effort [33,34,35].

Even though automatic tools can help transform local test codes to standard terminology, expert human review is still needed to solve complex issues. In our project, we used multiple approaches, including automated tools plus human expert suggestions, to correctly map 20 pathogens. The findings of our study showed that most of the hospitals faced LOINC System related problems. We analyzed all FAQs, categorized problems, suggested how to map accurately, and provided guidelines. In this process, we observed an improvement in LOINC mapping over time, especially in those hospitals who joined earlier. Indeed, our model helped develop a common infrastructure to increase the adaption of LOINC coding and reduce the technical barriers to implementing interoperability standards. All the participating hospitals used standardized vocabulary to identify laboratory tests; therefore, information exchange among hospitals would be bi-directional, improve scalability, and facilitate data compilation and access to patients’ longitudinal data. Furthermore, nationwide adoption of LOINC codes will help to reduce healthcare costs and improve quality of care by reducing erroneous and duplicate laboratory tests.

**Figure 7:**
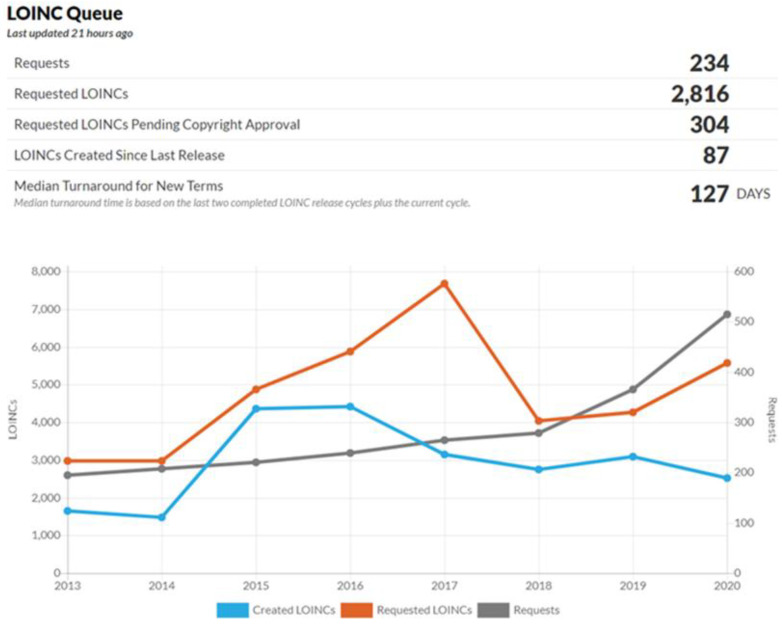
Frequency of LOINC additions.

Regenstrief has started updating LOINC and releasing it twice per year since 2010. LOINC has already increased to nearly 95,000 terms (laboratory terms: 57,817; clinical terms: 37,078) in their current version, which is approximately a 1.6-fold increase in the last decade. Regenstrief has also designed a system for requesting new terms or changes to the existing LOINC contents (Figure 7).

Over the study period, we taught clinicians and laboratory personnel how to use the updated LOINC terms. Taiwan’s NNDSS continues its work, and TCDC has added coronavirus as a reportable pathogen as of November 2020. To date, the participants include 63 hospitals, 20 medical centers, and 43 regional hospitals or district hospitals.

Strengths: Our study has several strengths. First, this was a nation-wide LOINC mapping project and evaluated the performance of LOINC mapping for 20 pathogens. Second, the performance of LOINC mapping improved significantly by using our multi-stage approach. Third, we provided new guidelines for LOINC mapping and solved complex LOINC mapping problems through discussion with the LOINC committee.

Limitations: Our study has several limitations that need to be addressed. First, our study included a limited number of hospitals and pathogens. However, more hospitals and pathogens have been included in the second phase of this project. Second, the use of LOINC mapping cannot provide guarantee for uniform interoperability of laboratory tests names and codes between different information systems, and our current study did not evaluate the feasibility of LOINC coding. Third, the challenges of LOINC mapping are geographically and chronologically diverse; mapping improvement through our model may not be representative of other health care systems.

Future work: The demand has been raised for health information exchange between interdepartmental in the same hospital or other hospitals to improve quality by sharing patients’ clinical information. In our current study, we only focused on the LOINC coding of 20 pathogens. In the future, we will focus on including more pathogens and managing new diseases such as COVID-19. Moreover, we will try to focus on profiling the microbiology data in FHIR for infection control, integrating biological results with other health information systems, and implementing existing standards in clinical data to support research. Finally, we will investigate the usability of LOINC, particularly with respect to its perceived benefits (e.g., interoperability, secondary data analysis).

## 5. Conclusions

Mapping local codes to standard terminology is always difficult, labor intensive, and requires domain expertise. However, it is important to exchange information among various departments in the same hospital or different hospitals. Our multi-stage approach improved LOINC mapping performance and provided guidelines that could help adoption of a new version in near future.

## Figures and Tables

**Table 1:** Notification conditions list.

Type	Pathogen Name	Background
Bacteria	*Campylobacter* species	Gram-negative bacterium, it is one of the most virulent zoonotic foodborne disease.
	*Mycobacterium tuberculosis* complex	*M. tuberculosis* can appear as either Gram-negative or Gram-positive. In Taiwan, tuberculosis is the most highly reported infectious disease. In 2016, there were 10,328 tuberculosis cases (43 cases per 100,000 population).
	*Listeria monocytogenes* (Listeriosis)	Gram-positive rod-shaped bacterium, it is a type of zoonotic foodborne disease. There were some outbreaks internationally during the last decade.
	*Salmonella* species	Gram-negative rod-shaped bacterium, it is one of the most virulent zoonotic foodborne disease. In 2014, there were 32 cases in Taiwan.
	*Streptococcus agalactiae* (group B strep, GBS)	Gram-positive spherical bacterium, group B strep disease is a common cause of severe infection in newborns.
	*Streptococcus pneumoniae*	Gram-positive bacterium, it can cause acute otitis media, pneumonia, bacteremia, and meningitis.
	*Streptococcus pyogenes* (group A strep, GAS)	Gram-positive bacterium, *S. pyogenes* can cause scarlet fever in children.
	*Vibrio parahaemolyticus*	Gram-negative halophilic bacterium, it is a type of foodborne illness. In 2014, there was an outbreak (66 cases) in Taiwan.
	*Yersinia enterocolitica* (Yersiniosis)	Gram-negative bacillus-shaped bacterium, infections of this strain are occasionally associated with eating raw or undercooked pork.
Viruses	Adenovirus	A genus of DNA viruses. There are 57 accepted human adenovirus types in seven species. They are usually associated with respiratory illnesses or conjunctivitis. We noted the enteric adenovirus types 40 and 41, which usually occur in children.
	Enterovirus	A genus of RNA viruses. We noted the non-polio enteroviruses that can cause disease in humans: 23 Coxsackie A viruses, six Coxsackie B viruses, 28 echoviruses, and five other enteroviruses, especially enterovirus A71, which is usually found in infants and young children.
	Hepatitis A virus (HAV)	A genus of RNA viruses. HAV is usually transmitted person-to-person through the fecal–oral route or consumption of contaminated food or water.
	Hepatitis B virus (HBV)	It is a genus of DNA viruses. HBV is transmitted when blood, semen, or another body fluid from a person infected with the HBV enters the body of someone who is not infected. Chronic Hepatitis B can lead to serious health issues, such as cirrhosis or liver cancer.
	Hepatitis C virus (HCV)	It is a genus of RNA viruses. Most people become infected with the Hepatitis C virus by sharing needles or other equipment to inject drugs.
	Herpes Simplex Virus (HSV)	It is a genus of DNA viruses. There are two types, HSV-1 and HSV-2, which are transmitted by contact with an infected person who has reactivation of the virus.
	Influenza virus	A genus of RNA viruses. There are four types of influenza viruses: A, B, C, and D. We noted the human influenza A and B viruses, which cause seasonal epidemics of disease almost every winter. Influenza is a contagious respiratory illness caused by influenza viruses that infect the nose, throat, and sometimes the lungs.
	Norovirus	It is a genus of RNA viruses. Norovirus is a very contagious virus that causes vomiting and diarrhea. In 2017, 108 cases were observed in Taiwan.
	Parainfluenza virus	It is a genus of RNA viruses. Human parainfluenza viruses (HPIVs) cause respiratory illnesses in infants and young children.
	Respiratory syncytial virus (RSV)	It is a genus of RNA viruses. RSV can spread when an infected person coughs or sneezes, and it is the most common cause of bronchiolitis and pneumonia in infants and older adults.
	Rotavirus	It is a genus of RNA viruses. Rotavirus spreads easily through the fecal–oral route in infants and young children.

**Table 2:** Performance of LOINC mapping in homegrown and outsourced systems.

Mapping Work	LOINC Mapping Completeness *	Hospital Numbers	Mapping to LOINC Codes (%) **	Unmapped Local Codes (%)
Homegrown	<60%	1	58%	42%
	60–70%	3	68%	32%
	70–80%	8	76%	24%
	80–90%	4	83%	17%
	>90%	10	96%	3%
Outsourced	<60%	2	25%	71%
	60–70%	7	67%	32%
	70–80%	2	73%	28%
	80–90%	1	85%	13%
	>90%	15	96%	3%

* Completeness: What extent have local codes been mapped to LOINC codes; ** The percentage was calculated on week 48 in 2016.

**Table 3:** Rate of LOINC mapping completeness based on experience.

LOINC Experience	Hospital Numbers	LOINC Mapping Completeness *
Yes	6	96.0% (SD: 0.034)
No	45	79.8% (SD: 0.178)

* Completeness: What extent have local codes been mapped to LOINC codes, and the percentage was calculated on week 48 in 2016.

**Table 4:** Suggestions for LOINC mapping problems.

Step	Process/Items	Suggested Mapping/Results
Implementation of ELR	Clarifying mapping style	Variable-style (testing a specific thing) and value-style (testing unknown results) should not be altered
	The real specimen	The real specimen for the specific laboratory tests, e.g., a blood test was divided into the tube and centrifuged to serum/plasma and not centrifuged to whole blood
	The test kit instructions	Obtain the method information from the kit instruction manual, for instance, that the immunoassays were enzyme-linked or fluorescence-linked
	The presentation of laboratory test results	Understand whether a quantitative or qualitative result from instrumentation will be produced
Audit LOINC mapping	Viral hepatitis	The majority of mapping styles were variable-style names; the LOINC System was centrifuged to serum or plasma; the LOINC Method was immunoassays; and the result for identifying the positive or negative of a specific organism Suitable mapping: LOINC Property “ACnc” and LOINC Scale “Ord”
	Mycobacterium	The most common mapping style was variable-style names; the specimen was varied from human body; the method was decided by test combinations including “acid-fast stain” and “organism specific culture”; and the result for “acid-fast stain” Suitable mapping: LOINC Property “Prid” and LOINC Scale “Ord” AND ……; the result for “organism specific culture” Suitable mapping: LOINC Property “ACnc” and LOINC Scale “Ord”
	Antigen or antibody tests for non-mycobacterium	The majority mapping style was also variable-style names; the specimen was varied by different conditions; the LOINC Method might refer to the test kit about immunoassays, immunofluorescence, latex agglutination assay and so forth; and the result for identifying the positive or negative of a specific organism Suitable mapping: LOINC Property “ACnc” and LOINC Scale “Ord”
	Antigen or antibody tests for non-viral hepatitis	The most common mapping style was also variable-style names; the specimen was varied by different conditions; the LOINC Method might be immunoassays for antigen or antibody tests, and the LOINC Method might be “Probe.amp.tar” for PCR (polymerase chain reaction) tests; and the result for identifying the positive or negative of a specific organism Suitable mapping: LOINC Property “ACnc” and LOINC Scale “Ord”
	Viral/bacterial cultures	The majority mapping style was value-style names; the specimen was varied from human body; the LOINC Method was separated into “Culture” for all pathogens, “Aerobic culture” for aerobic bacteria, and “Anaerobic culture” for anaerobic bacteria; and the result for identifying the absence, or if present, Suitable mapping: LOINC Property “Prid” and LOINC Scale “Nom”
Evaluated reported data	Correctness of LOINC six parts	Laboratory test name map to LOINC Component: Anti-HBc was mapped to “Hepatitis B virus core Ab” Anti-HBc IgG was mapped to “Hepatitis B virus core Ab.IgG” Influenza A/B Viruses Antigen Rapid Test was mapped to “Influenza virus A & B Ag” Laboratory test specimen map to LOINC System: Nasal swab was mapped to “Nose”; Nasopharyngeal swab was mapped to “Nph”; Rectal swab was mapped to “Anal”; Bronchial washing was mapped to “BAL” Laboratory method map to LOINC Method: Chemiluminescent immunoassay or lateral-flow immunoassays was all mapped to “EIA”, and real-time PCR was mapped to “Probe.amp.tar” Laboratory test result map to LOINC Property and LOINC Scale: If the result was presence or not, it was mapped to “Pr/Ord”, if positive or not, it was mapped to “ACnc/Ord”; The result as reporting name was mapped to “Prid/Nom”, the quantitative result was mapped to “ACnc/Qn”
	Usefulness of LOINC code	For the quantitative result of Anti-HCV by EIA, Suitable map: LOINC 5198-7 (Hepatitis C virus Ab: ACnc: Pt: Ser: Qn: EIA) For the qualitative result,Suitable map: LOINC 13955-0 (Hepatitis C virus Ab: ACnc: Pt: Ser/Plas: Ord: EIA) For the rank result of acid-fast stain in bronchial washing by Ziehl-Neelsen, Suitable map: LOINC 76083-5 (Microscopic observation: Pr: Pt: BAL: Ord: acid-fast stain. Ziehl-Neelsen) For the qualitative result of Tuberculosis (TB) culture in pleural effusion, Suitable map: LOINC 53909-8 (Mycobacterium sp. identified: Prid: Pt: Plr fld: Nom: Organism specific culture)
	Completeness (coverage) of the test of all required fields	Total mapped: 81.8% Total unmapped: 17.7% Undecided/to be confirmed: 0.5%

**Table 5:** Compression of mapping performance before and after project.

Mapping Issues	Before LOINC 2.50	After LOINC 2.50	Review in LOINC 2.64
1. LOINC *Method*: acid-fast stains, its result is “1+, 2+, 3+”, LOINC *Scale*: Ord	1 code: 72357-7	LOINC 2.58 Guideline: acid-fast stains, the LOINC *Component* is microscopic observation	Add 5 new codes: 88171-4/88172-2/88173-0/88234-0/88366-0
2. LOINC *Method*: rapid immunoassay in Influenza virus	3 codes: 72356-9/72366-8/72367-6	LOINC 2.56 Guideline: changed the name of the EIA method to IA (and EIA.rapid to IA.rapid)	Add 3 new codes: 80381-7/80382-5/80383-3
3. LOINC *Method*: EIA, Hepatitis B virus core Ab.IgG+IgM in Ser	1 code: 51914-0 LOINC *Method* is not specified		Add 1 new code: 83100-8 LOINC *Method* by IA
4. LOINC *Method*: EIA or LA, Streptococcus pneumoniae Ag in Urine	1 code: 24027-5 LOINC *Method* is not specified		Add 1 new code: 77949-6 LOINC *Method* by IA.rapid
5. LOINC *Method*: EIA, Adenovirus Ag in Nose	1 code: 43614-7 LOINC *Method* is not specified		Add 2 new codes: but also change the LOINC *System* 88603-6 LOINC *System* in Respiratory lower, LOINC *Method* by IA 88602-8 LOINC *System* in Nph, LOINC *Method* by IA
6. LOINC *Method*: anaerobic + aerobic culture, bacteria identified in Bld	3 codes: 17928-3 LOINC *Method* by aerobic culture 17934-1 LOINC *Method* by anaerobic culture 600-7 LOINC *Method* by culture	Guide for Using LOINC Microbiology Terms: LOINC *Method*: culture, which is a generic *Method* that could encompass any of the other culture types	
7. LOINC *System* in Thrt, Influenza virus A RNA by Probe.amp.tar	2 codes: 76077-7 LOINC *System* in BAL 76078-5 LOINC *System* in Nph		Add 2 new codes: 85477-8 LOINC *System* in Respiratory.upper 88599-6 LOINC *System* in Respiratory.lower
8. LOINC *System* in sputum or Nph, RSV Ag by IF	2 codes: 5875-0 LOINC *System* in Thrt 32040-8 LOINC *System* in nose	2 codes: 77389-5 LOINC *System* in BAL 77390-3 LOINC *System* in Nph	Add 1 new code: 88909-7 LOINC *System* in Respiratory.lower
9. LOINC *Property* and LOINC *Scale*: microscopic observation in sputum by acid-fast stain. Kinyoun, its result is “1+, 2+, 3+”, LOINC *Scale*: Ord	1 code: 645-2, LOINC *Property* is Prid, and LOINC *Scale* is Nom	Sample results can be ranked, such as 1+, 2+, 3+ (LOINC *Scale* Ord), and the kind of Property will usually be presence (PrThr, for results based on the presence/absence of an analyte regardless of whether an internal cut off value is used to determine the ordinal result)	Add 1 new code: but change the LOINC *System*: 88631-7 LOINC *System* in Respiratory.lower
10. LOINC *Property* and LOINC *Scale*: *Streptococcus agalactiae* in cervix by organism specific culture, its result is “presence/absence”, LOINC *Property*: ACnc	1 code: 581-9 LOINC *Property* and LOINC *Scale*: ACnc/Ord	LOINC 2.56 Guideline: All of the existing terms with a Property of ACnc, Pr or Threshold and a Scale of Ord were updated to have the Property PrThr	1 code: 581-9 LOINC *Property* and LOINC *Scale*: PrThr/Ord

**Table 6:** Experts’ responses related to FAQs.

FAQs	Question	Answer
LOINC knowledge	*“What is the meaning of the LOINC Class?”*	*“LOINC Class is a categorical classification for a LOINC term. The 17 categories are relatively broad and are intended to make it easier to sort and browse the database for users in LOINC 2.50”.*
Notification principle	“In *one specimen, different kinds of pathogens were detected?*”	“*The notification is based on the positive result, therefore you need to report different pathogens which we are monitoring in this case.*”
LOINC domain	“*In microbiology, users are always confused about the test order and how to report.*”	“*Users need to think about the kind of laboratory tests. The mapping style could be divided into- Variable-style names: testing specific thing/quantitative/positive or negative; Value-style names: testing unknown results, like culture results/qualitative/pathogen’s name*.”

## Supplementary Materials

The following are available online at https://www.mdpi.com/article/10.3390/diagnostics11091564/s1. Table S1: Description of LOINC six axes; Table S2: Inceptive provided by government.
