# Supplementary material for: Logical Observation Identifiers Names and Codes (LOINC®) Applied to Microbiology: A National Laboratory Mapping Experience in Taiwan
Source: Diagnostics (Basel). 2021 Aug 28;11(9):1564. doi: 10.3390/diagnostics11091564 (PMC8464801; doi:10.3390/diagnostics11091564)
Supplement: Supplementary file 1 [file diagnostics-11-01564-s001.zip › diagnostics-1251951-supplementary.pdf]

Supplementary Table S1: Description of LOINC six axes.

Axis	Meaning
Component	What is being measured
Property	The characteristic of how it is being measured
Timing	When the measurement is being completed
System	Where the analyte originates
Scale	Which way will the test result be expressed
Method (optional)	What method was used to make this measurement

Supplementary Table S2: Inceptive provided by government

Year	Participant	Financial support
**2014**	medical centers: 15<br>Regional hospital: 5	NT$ 0.6~0.8 million per hospital
**2015**	medical centers: 2<br>Regional hospital: 7	NT$ 0.6~0.75 million per hospital
**2016**	medical centers: 2<br>Regional hospital: 22	NT$ 0.48~0.58 million per hospital
